# Book Review

**DOI:** 10.1120/jacmp.v7i4.2420

**Published:** 2006-11-28

**Authors:** Paul S. Cho

**Affiliations:** ^1^ Department of Radiation Oncology University of Washington 1959 NE Pacific Street Seattle Washington 98195‐6043

## Abstract

*Advances in Medical Physics 2006*, Edited by Anthony B. Wolbarst, Robert G. Zamenhof, and William R. Hendee, Medical Physics Publishing, Madison, Wisconsin 2006, 331 pp ISBN: 978‐1‐30524‐34‐,7 List price: $80

From the word “Advances” in the title, one might expect the subject matters to be exclusively in the category of recent development. However, a quick review reveals that the content is a mixture of both basic and current topics. In fact, some chapters devote more pages to the description of fundamental principles than to advanced technologies. So why the title? According to the preface, *Advances in Medical Physics 2006* is the inaugural volume of a biennial series designed to provide reviews of current technologies in medical physics. The main focus of this initial volume is to provide up‐to‐date scientific and technical background information. As such, some elementary concepts essential to understanding of individual subspecialties are presented.

The book is written by 27 contributing authors and is illustrated with 211 figures and 21 tables. The references are up to date. The chapters address primarily the medical imaging technologies and are organized as follows:

1. Digital Radiography and Fluoroscopy
2. Mammography and Other Breast Imaging Techniques
3. Computed Tomography
4. Nuclear Medicine
5. Magnetic Resonance Imaging
6. Medical Ultrasonic Imaging
7. Molecular Imaging
8. Overview of Medical Imaging Informatics
9. Evolving and Experimental Technologies in Medical Imaging
10. Biological Effects of Low Doses of Ionizing Radiation
11. Radiation Therapy
12. Magnetic Nerve Stimulation

In general, each chapter begins with background/historical materials followed by an overview of current topics. Because of the generous allocation to background information, this original issue will be of interest to those who are not yet familiar with or looking to refresh themselves in basic concepts. Well‐illustrated primers are found in chapters on Digital Radiography, CT, MRI, and Ultrasound.

Practicing medical physicists will also find the book useful as it contains updated information on techniques, device specifications, and theories. Where applicable, the book also addresses QA related issues. Overall, the articles will provide a timely survey of what is happening in medical physics subspecialties (other than your own perhaps). Some of the recent topics found in the chapters are: ECG‐gated cardiac CT, contrast mechanisms for functional MRI, powermode Doppler, digital mammography, nanoparticles for molecular imaging, intra‐fraction motion in radiotherapy, active matrix flat‐panel imagers, electronic medical records, BEIR VII (2006) consensus on linear, no‐threshold dose‐response model, bystander effect, genomic instability, etc. The chapter on “Evolving and Experimental Technologies in Medical Imaging” has more of a futuristic orientation and reports on interesting new technologies such as optical and near‐infrared imaging, terahertz imaging, microwave imaging, electric impedance tomography, magnetoencephalography, and magnetocardiography. Do not expect to find in‐depth discussions of these advanced topics. But to those looking for a quick overview, the chapter provides concise and sufficiently detailed summaries of novel technologies that are undergoing rapid development. Each chapter ends with a summary section which includes discussions on current trends, issues, and future directions. Some of the commentaries by the experts are insightful and help one gain an informed perspective on the ever evolving medical physics technologies. One shortcoming of the book is the lack of a subject index, which somewhat diminishes its utility. In order to find a specific topic and all its occurrences, one would have to browse through multiple chapters. For example, discussions on “tomosynthesis” appear in chapters 1, 2, and 9.

In summary, the book is generally well written and will be beneficial to both students and professionals who want a broad overview of the current technologies. Those not interested in background information might pass on this initial volume and wait for the future issues, which presumably will contain less background materials and more recent developments in principal areas of medical physics, along with specific topics such as ultra‐high frequency ultrasound, biophysical processes underlying for MR relaxation, and image‐guided radiation therapy.

